# Intrathyroidal Thymic Carcinoma With Arterial Invasion and Presumed Poor Tolerance to Cerebral Ischemia Safely Treated With Intraoperative Carotid Artery Protection With a Covered Stent: A Case Report

**DOI:** 10.1002/ccr3.70934

**Published:** 2025-09-23

**Authors:** Masato Nagaoka, Naoki Toya, Eisaku Ito, Miku Maeda, Michiyasu Fuga, Yosuke Mizunari, Takao Ohki

**Affiliations:** ^1^ Department of Otorhinolaryngology The Jikei University School of Medicine Minato‐ku Tokyo Japan; ^2^ Department of Surgery The Jikei University School of Medicine Minato‐ku Tokyo Japan; ^3^ Department of Pathology The Jikei University School of Medicine Minato‐ku Tokyo Japan; ^4^ Department of Neurosurgery The Jikei University School of Medicine Minato‐ku Tokyo Japan

**Keywords:** brain ischemia, carotid arteries, stents, thymic carcinoma, thyroid neoplasms

## Abstract

This report demonstrates the effectiveness of covered stents in surgery for a patient with thyroid cancer suspected of carotid artery invasion and poor tolerance to cerebral ischemia. Covered stents allowed safe management of blood vessels while maintaining blood flow, potentially expanding their applicability in head and neck tumor surgery.

## 1 Introduction

Potential tumor invasion of large blood vessels is a concern for surgeons due to the risk of major bleeding. Particularly, management of thyroid cancer that invades the carotid artery is a major challenge for head and neck surgeons, as it can lead to cerebral infarction or death during surgery.

When performing surgery on lesions suspected of carotid artery invasion, the patient's tolerance to cerebral ischemia should be determined. Based on these results, vascular replacement or ligation must be carefully considered [1].

A definitive preoperative diagnosis of intrathyroidal thymic carcinoma (ITTC) is challenging, and the condition in most cases is assessed as poorly differentiated thyroid carcinoma (PDTC). Typically, PDTC progresses at a faster rate than differentiated thyroid cancer and may increase in size before surgery [2].

This case report describes a patient with ITTC suspected of carotid artery invasion and poor tolerance to cerebral ischemia. This is the first report of a case in which we decided to use a covered stent intraoperatively, and in collaboration with a vascular surgeon, the tumor was safely removed without complications.

## 2 Case History/Examination

**FIGURE 1:**
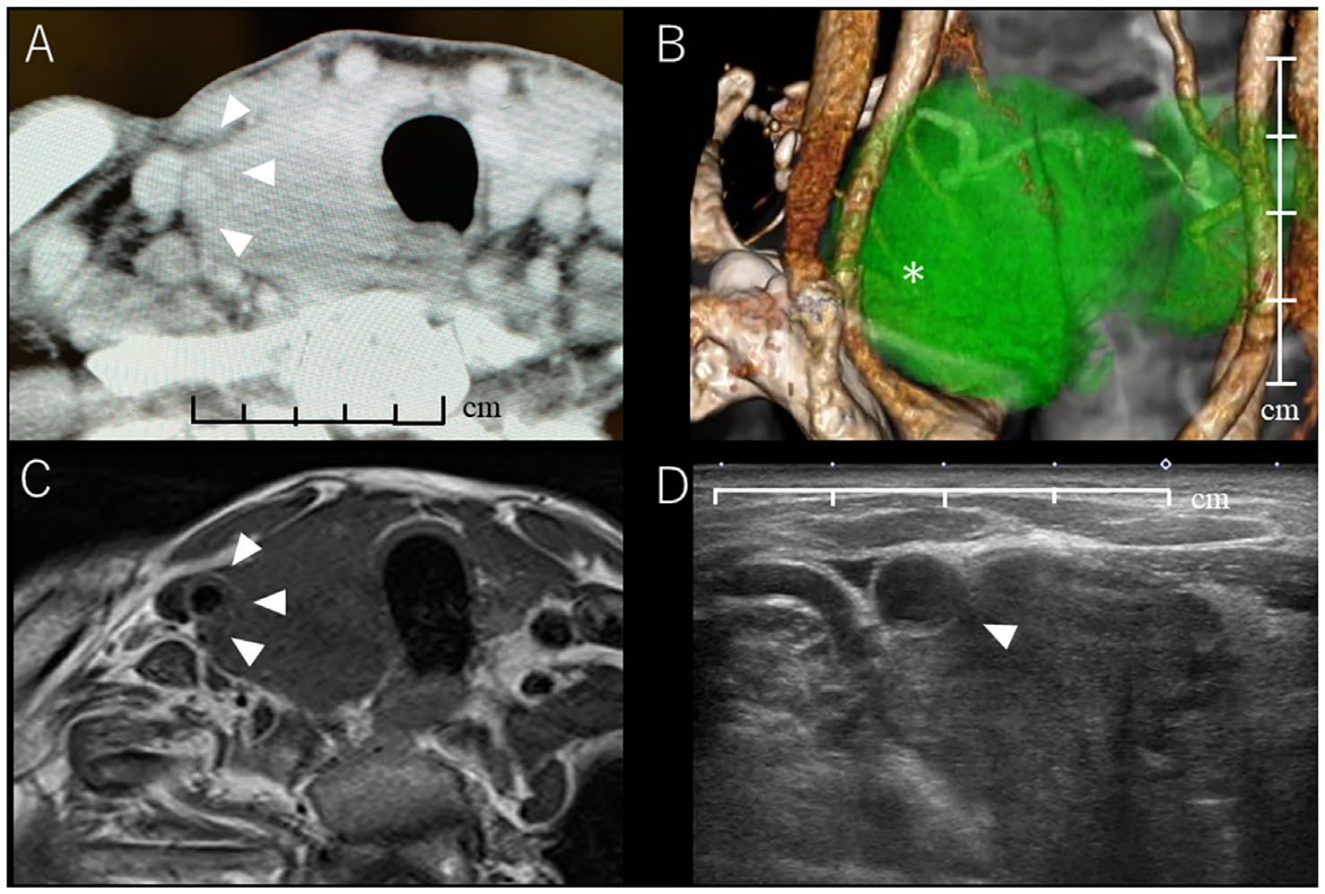
Preoperative images. (A) Preoperative CT axial image. The tumor was in contact with the common carotid artery for half of its circumference. The boundary was unclear in some areas (arrowhead). (B) 3D CT image. The common carotid artery was compressed by a tumor (asterisk), which appears to partially surround it. (C) T1‐weighted MRI image. Similarly, the area between the tumor and blood vessels is unclear (arrowhead). (D) Ultrasound image. The boundary between the outer membrane and middle membrane of the common carotid artery and the tumor is partially unclear (arrowhead). 3D, three dimensional; CT, computed tomography; MRI, magnetic resonance imaging.

A 76‐year‐old man visited the hospital complaining of neck pain that had persisted for 2 months. He was referred to our hospital for further examination and treatment after being diagnosed with a thyroid tumor at another hospital. Cytological examination indicated PDTC. Preoperative imaging included computed tomography (CT), magnetic resonance imaging (MRI), and ultrasound (US), which suggested that the tumor encircled 180° of the area and extended partially to the outer and middle membranes (Figure 1A–D). Additionally, the tumor was 40 × 35 × 30 mm in size.

**FIGURE 2:**
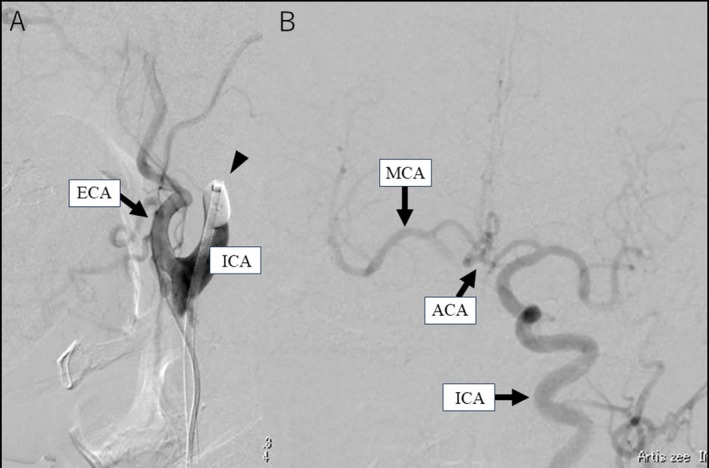
Evaluation of cerebrovascular collateral circulation using balloon occlusion tests. (A) Balloon occlusion test (lateral view): Right common carotid angiography shows the right internal carotid artery is occluded using a balloon (arrowhead), effectively blocking blood flow in the internal carotid artery. Only the external carotid artery is opacified (arrow), confirming accurate occlusion. (B) Balloon Matas test (frontal view): Carotid angiography shows the presence of collateral blood flow to the right internal carotid via the anterior communicating artery. However, visualization of key perforating arteries such as the lenticulostriate and anterior choroidal arteries were insufficient. The front view is on the right side of the image on the left and on the left side of the image on the right. The lateral view is the face side on the left side of the image and the occipital side on the right side. ACA, anterior communicating artery; ECA, external carotid artery; ICA, internal carotid artery; MCA, middle cerebral artery.

The cancer may have been poorly differentiated; therefore, clearly classifying the stage was impossible. However, since the tumor was wrapped around half of the carotid artery, we chose to perform a total thyroidectomy and postoperative iodine therapy. Considering how to handle the blood vessels during surgery is crucial since the tumor was in contact with the blood vessels at 180°. A preoperative balloon occlusion test was conducted under local anesthesia to assess the risk of cerebral ischemia associated with right internal carotid artery injury. Following balloon occlusion of the right internal carotid artery (Figure 2A), left internal carotid angiography demonstrated collateral blood flow to the right internal carotid artery through the anterior communicating artery, with insufficient visualization of perforating arteries, including the lenticulostriate and anterior choroidal arteries (Figure 2B). The patient exhibited sensory abnormalities in the contralateral lower extremity within 2 min and 30 s of balloon occlusion. These findings indicated a low tolerance for cerebral ischemia during occlusion of the right internal carotid artery.

## 3 Methods

Considering the patient's limited ischemic tolerance, the decision was made intraoperatively to protect the right common carotid artery with a covered stent.

**FIGURE 3:**
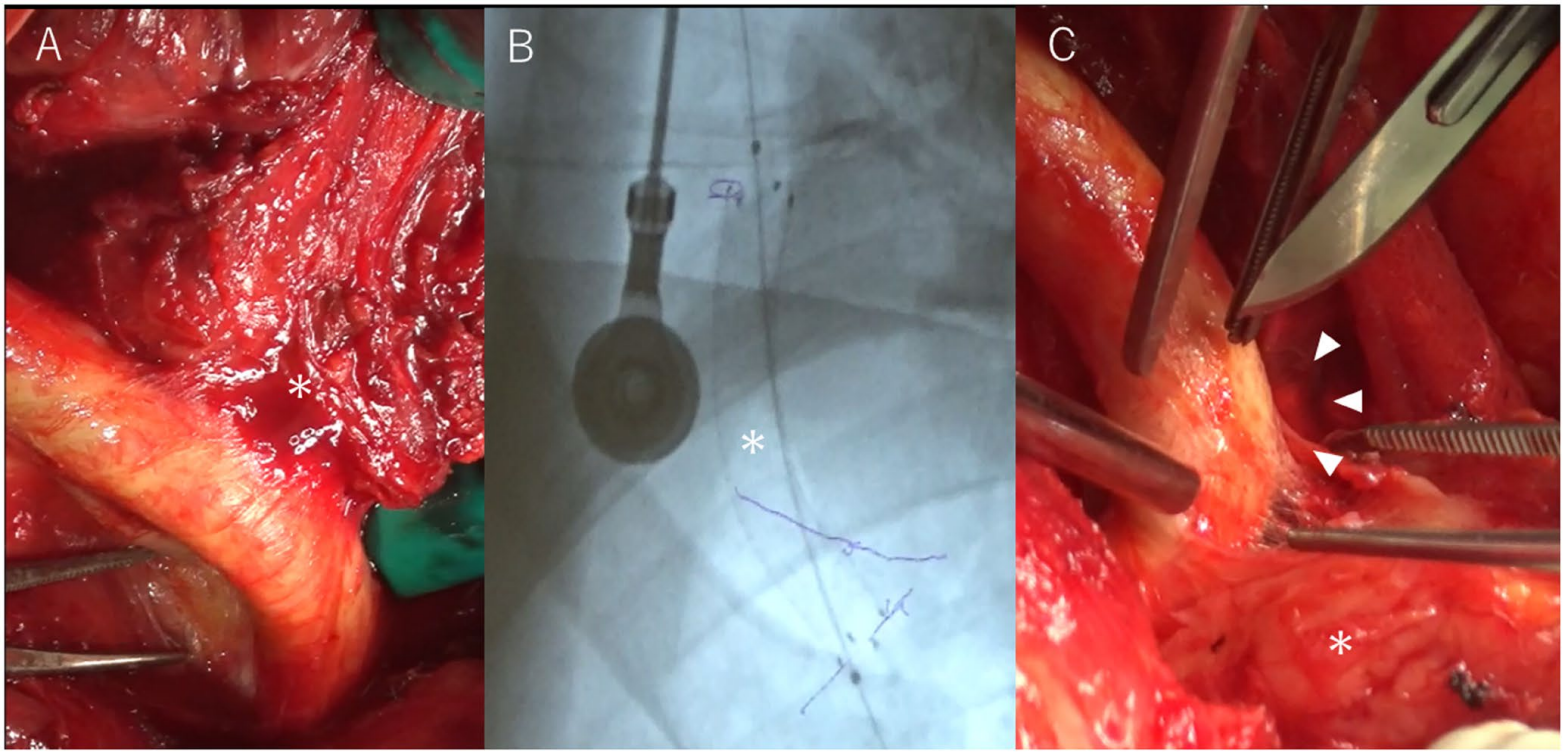
Intraoperative findings. (A) The intraoperative findings of the common carotid artery and the tumor (asterisk) were revealed. The carotid artery was in contact with the tumor at 180°. (B) Fluoroscopic image of the surgical field revealing the inserted covered stent (asterisk). (C) The tumor was safely separated from the carotid artery (arrowhead).

The surgery was performed in collaboration with a vascular surgeon. Under general anesthesia, a J‐shaped incision was made in the neck and the left thyroid lobe was dissected from the trachea. Since the area around the brachiocephalic artery below the tumor could not be clearly visualized due to tumor invasion, the sternocleidomastoid muscle was cut off at the clavicle, and the anterior cervical muscles were resected. The internal jugular vein and common carotid artery were identified above and below the tumor location. We attempted to dissect the hard tumor from the common carotid artery; however, this could have damaged the arterial wall. Therefore, a 7Fr sheath was inserted into the common carotid artery (CCA) under direct visualization and retrogradely placed a 5–50 mm Viabahn stent graft (W.L. Gore & Associates, Flagstaff, Arizona, USA) in the proximal CCA. During surgery, 5000 IU of heparin was administered. After the insertion of a covered stent, the tumor was safely detached from the carotid artery (Figure 3A–C). Near‐infrared cerebral oxygen monitoring was performed during the operation. The recurrent laryngeal nerve was also resected, and the procedure was completed with an end‐to‐end nerve anastomosis using the ansa cervicalis. No evidence of tracheal invasion was found. Finally, a paratracheal lymph node dissection was performed. In addition to performing recurrent laryngeal nerve reconstruction during surgery, dual antiplatelet therapy (DAPT) was required early postoperatively; consequently, a tracheal fenestration was performed, considering the risk of hematoma and laryngeal edema. Notably, no complications, such as cerebral infarction, occurred. The patient was started on 100 and 75 mg/day of aspirin and clopidogrel, respectively, from the third postoperative day. However, on postoperative day 6, the neck wound became infected, and antibiotic therapy was initiated. On postoperative day 13, a procedure was performed to drain the pus, and the patient was ultimately discharged on postoperative day 22.

## 4 Conclusion and Results

**FIGURE 4:**
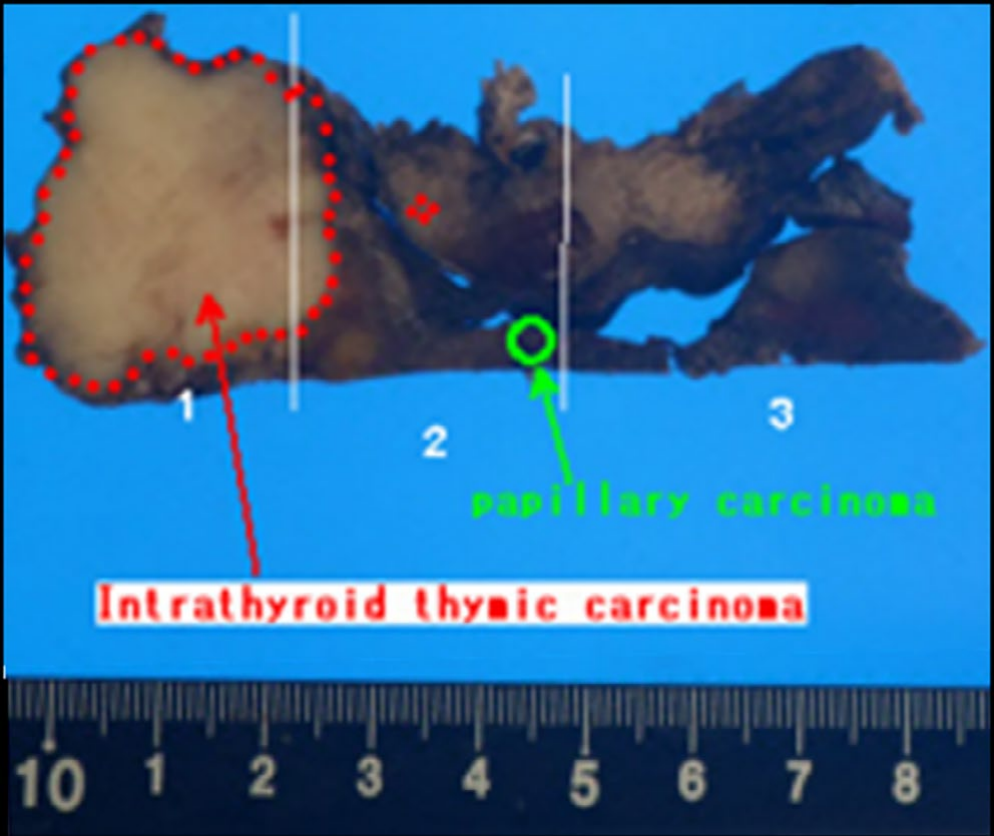
The resected specimen. Histological examination revealed that no elastic fibers were identified in the carotid artery, and no invasion of the media was observed, at least in the carotid artery. A small nodule (3.5 × 3.5 mm) of papillary carcinoma was found in the isthmus of the thyroid gland, which was not continuous with the main lesion (intrathyroid thymic carcinoma).

**FIGURE 5:**
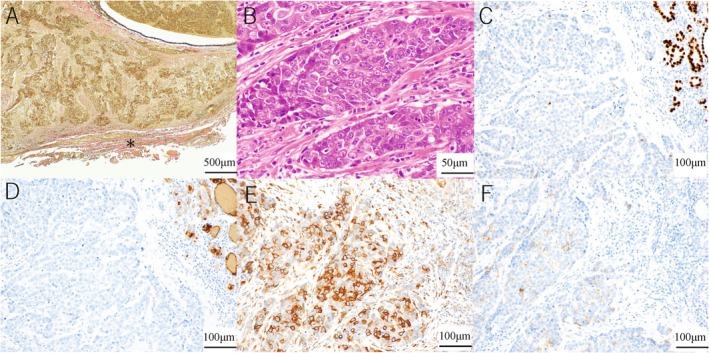
Histopathological image of resected tumor specimen. (A) Histopathological image (elastica van gieson staining). The elastic fibers of the carotid artery could not be identified, and infiltration of the carotid artery remained unclear (asterisk). (B) Histopathological image (hematoxylin staining). The magnified image revealed no nuclear grooves or intranuclear inclusions, and the tumor was poorly differentiated. (C) Immunohistochemical analysis revealed that the tumor cells were negatively reactive to thyroid transcription factor‐1 (TTF‐1). (D) Immunohistochemical analysis revealed that the tumor cells were negatively reactive to thyroglobulin. (E) Immunohistochemical analysis revealed that the tumor cells were positively reactive to CD5. (F) Immunohistochemical analysis revealed that the tumor cells were positively reactive to CD117.

**FIGURE 6:**
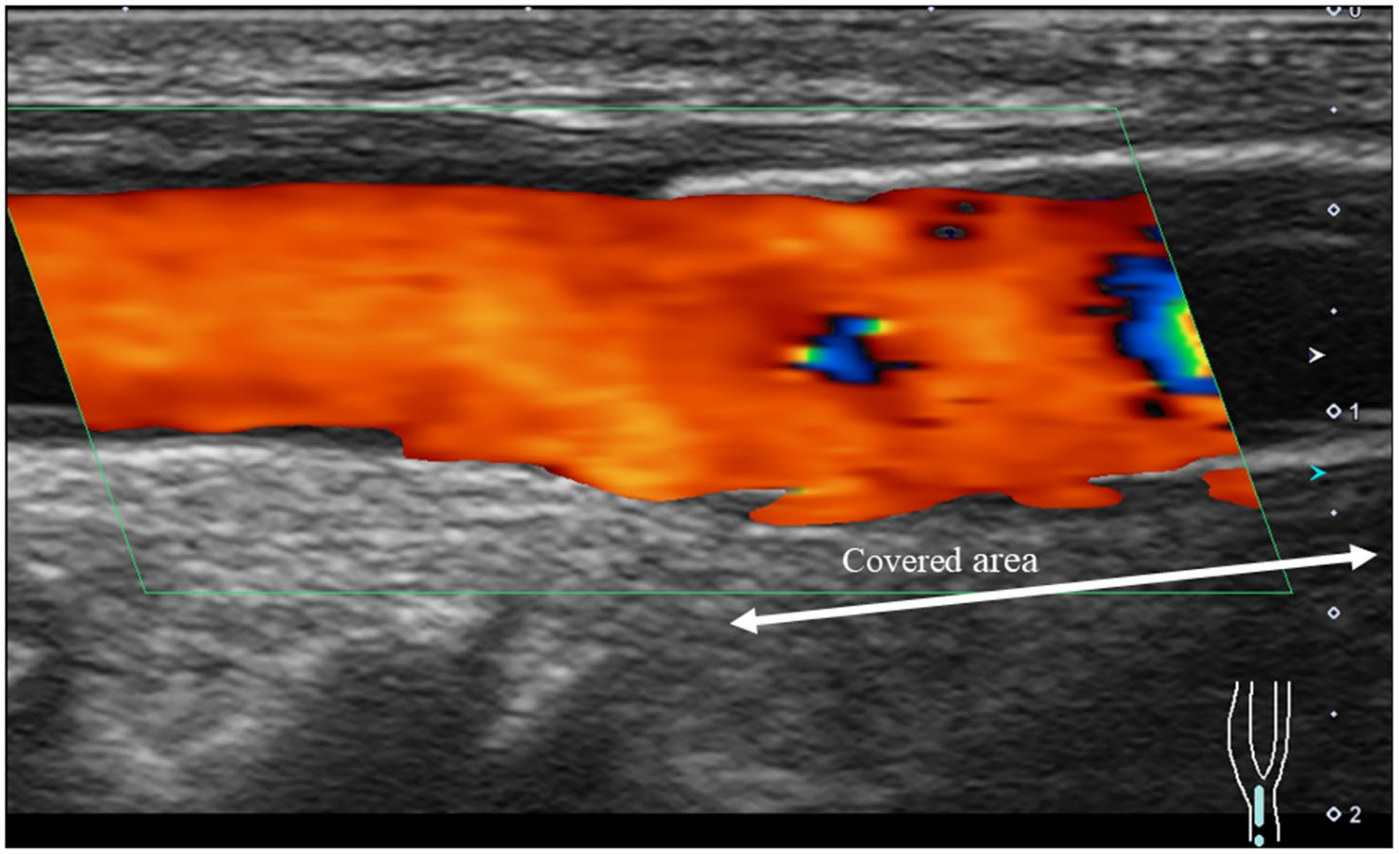
Condition of covered stent 4 years after surgery. Ultrasound image. The blood flow was maintained inside the stent.

We safely performed thyroid surgery using a covered stent in patients who were not ischemia‐resistant. Histological examination revealed that no elastic fibers were identified in the carotid artery, and no invasion of the media was observed, at least in the carotid artery. A small nodule (3.5 × 3.5 mm) of papillary carcinoma was found in the isthmus of the thyroid gland. No continuity with the main lesion was observed, and it was believed to be a multiple lesion (Figure 4). No signs of papillary or follicular carcinoma in the main lesion were found, and the tumor was poorly differentiated. Undifferentiated carcinoma and metastatic squamous cell carcinoma were listed as the top differential diagnoses (Figure 5A,B). Immunohistochemical analysis revealed that the tumor cells were negative for thyroid transcriptase factor‐1 (TTF‐1) and thyroglobulin, while positive for CD5 and CD117 (Figure 5C–F). Based on these findings, the final histopathological diagnosis was ITTC. We performed radioactive iodine (RAI) therapy due to a positive microscopic margin of invasion into the pretracheal muscle. The patient has remained free of recurrence for 4 years. The stent remains in place without any complications, and ultrasound evaluation confirms that internal blood flow is being maintained (Figure 6).

## 5 Discussion

Differentiated thyroid cancer centered on papillary carcinoma can be peeled off by shaving, and the recurrence rate is low [3]. A previous report also suggests a relationship between advanced thyroid cancer and large blood vessels, proposing that combined resection of the tumor and the adventitia of the carotid artery may be feasible in most cases [4]. However, deaths from postoperative bleeding due to carotid artery fragility have raised concerns about the reliability and safety of this method. To reduce this risk, various vascular interventions have been explored for head and neck cancer with large vessel invasion [1, 5]. In particular, MRI often reveals that lesions in contact with the carotid artery at 180° or more exhibit parenchymal invasion, necessitating surgical preparation for the blood vessels [6].

In this case, the preoperative cytological diagnosis was PDTC, and the postoperative diagnosis was ITTC. PDTC represents an intermediate stage in the progression from well‐differentiated thyroid cancer to anaplastic (undifferentiated) thyroid carcinoma. However, no clear guidelines are currently available for its treatment [7]. ITTC is difficult to diagnose preoperatively using US or CT, and does not reveal the typical histological features of papillary adenocarcinoma or follicular carcinoma. Few reports exist with evidence for a treatment method; however, this case demonstrates similar characteristics, and a definitive diagnosis was made based on positive immunostaining for TTF‐1 and CD5 in the resected specimen. The patient has been in remission for 4 years following the surgery and RAI [2].

In this case, the preoperative diagnosis of highly malignant poorly differentiated cancer, along with US and MRI findings, indicated the need for caution due to the possibility of vascular invasion. Furthermore, this case yielded a positive result on the preoperative balloon occlusion test, indicating that a brief period of blood flow interruption was not allowed. Covered stents are commonly used to prevent open carotid blowout syndrome, a potentially fatal condition resulting from the rupture of the carotid artery. There are also a few reports on the staged use of the device in the treatment of carotid body tumors and head and neck squamous cell carcinoma [8, 9, 10].

However, taking antiplatelet drugs is desirable in the early postoperative period after stent placement. DAPT is generally recommended for 6–12 months postoperatively, followed by single antiplatelet therapy [11]. Therefore, the risk of postoperative hematoma is a concern. Long‐term complications include stent occlusion, infection, and heparin‐induced thrombocytopenia (due to heparin coating). Therefore, regular follow‐up with ultrasound is necessary. While occlusion is a complication that can occur at any time, the most dangerous time is 6 months postoperatively.

Despite these disadvantages, we believe that the proactive use of covered stents in cases of suspected carotid artery invasion will enable safe removal of tumors while maintaining blood flow. This approach will reduce stress on the surgeon and broaden the range of indications.

## Author Contributions

**Masato Nagaoka:** conceptualization, data curation, formal analysis, investigation, methodology, project administration, resources, supervision, validation, visualization, writing – original draft, writing – review and editing. **Naoki Toya:** conceptualization, data curation, formal analysis, investigation, methodology, resources, supervision, validation, visualization, writing – review and editing. **Eisaku Ito:** data curation, formal analysis, validation, visualization, writing – review and editing. **Miku Maeda:** data curation, investigation, resources, validation, visualization, writing – review and editing. **Michiyasu Fuga:** data curation, investigation, resources, validation, visualization, writing – review and editing. **Yosuke Mizunari:** data curation, investigation, resources, validation. **Takao Ohki:** conceptualization, data curation, formal analysis, investigation, methodology, resources, supervision, validation, visualization, writing – review and editing.

## Consent

Written informed consent was obtained from the patient to publish this report in accordance with the journal's patient consent policy.

## Conflicts of Interest

Takao Ohki is a paid consultant for WL Gore.

## Data Availability

The datasets supporting the conclusions of this article are included within the article.
